# Association Between ABO Blood Group and Venous Thromboembolism Risk in Patients With Peripherally Inserted Central Catheters: A Meta-analysis and Systematic Review

**DOI:** 10.3389/fonc.2022.906427

**Published:** 2022-07-08

**Authors:** Qiang Zhang, Hong Peng, Lu Hu, Ran Ren, Xingqiao Peng, Jifang Song

**Affiliations:** Department of Oncology, Army Medical Center of PLA, Chongqing, China

**Keywords:** ABO blood group, venous thromboembolism, peripherally inserted central catheters, meta-analysis

## Abstract

**Background:**

Previous studies have evaluated the association between ABO blood group and venous thromboembolism (VTE) risk in patients with peripherally inserted central catheters (PICCs). However, it remains unclear whether ABO blood groups are associated with PICC-associated VTE risk. Therefore, we conducted a meta-analysis of related studies to elucidate the potential role of ABO blood group as a risk factor for PICC-associated VTE.

**Methods:**

All detectable case–control and cohort studies comparing the role of ABO blood group as a risk factor for PICC-associated VTE were collected for this analysis by searching PubMed, Embase, CNKI, Web of Science, and Wanfang. We conducted a meta-analysis of the eligible studies and computed the summary risk estimates with random or fixed effects models.

**Results:**

A total of four studies involving 7,804 patients were included. Meta-analysis of the studies showed that the risk of PICC-associated VTE was significantly higher in blood types A [odds ratio (OR)=1.54, 95% CI=1.17–2.03), p=0.002], B (OR=2.35, 95% CI=1.71–3.23, p<0.0001), and AB (OR=2.55, 95% CI=1.68–3.88, p<0.0001) and lower in blood types O (OR=0.58, 95% CI=0.45–0.74, p<0.0001). Subgroup analysis based on ethnicity demonstrated that blood type O may be a genetic protective factor for PICC-associated VTE in Asians. Among Caucasians, individuals with blood types B and AB have a higher risk of PICC-associated VTE. Blood types A, B, and AB are risk factors for PICC-associated VTE in Asians.

**Conclusions:**

Blood type O is associated with a decreased risk of PICC-associated VTE, especially in Asian populations. Moreover, blood types A, B, and AB are risk factors for PICC-associated VTE.

## Introduction

A peripherally inserted central catheter (PICC) is a central catheter that is inserted through a peripheral vein, such as the arm vein or saphenous vein, and tipped to the superior vena cava or right atrium (1–3). PICCs can be used for long-term chemotherapy drugs and extended antimicrobial therapy, total parenteral nutrition, or infusion of drugs that are not suitable for peripheral intravenous infusion (4). PICCs are popular because they are easy to insert and cost effective.

However, with the clinical popularity of PICCs, complications related to PICCs have also emerged (5, 6). PICC-associated venous thromboembolism (VTE) is considered one of the most common and serious complications (7). As an intravascular foreign body, PICC catheters directly damage the vascular intima, which is one of the major causes of VTE (8, 9). It has been reported that the incidence of PICC-associated VTE is 3%–20% (10, 11). Many studies have assessed factors that contribute to the formation of PICC-associated VTE, such as the catheter insertion method, catheter diameter, catheter tip location, catheter retention time, use of chemotherapy agents for malignancies, and obesity (12–16). These factors can provide a reference for the construction of a PICC-associated VTE prediction model.

It is well known that the ABO blood group has a significant effect on hemostasis by affecting the plasma level of coagulation factor VIII by affecting vascular Willebrand factor (vWF) (17). Many studies have found an increased risk of VTE in patients with non-O blood type (18–21). A meta-analysis by Dentali et al. showed that patients with VTE had a significantly higher prevalence of non-O blood type (22). However, it is not clear whether ABO blood group is a risk factor for PICC-associated VTE.

Currently, there have been studies on the association between ABO blood group and PICC-associated VTE susceptibility. Koo et al. found that the incidence of PICC-associated VTE was significantly increased in patients with blood type B (23). Blood types A and AB were not associated with the incidence of PICC-associated VTE. Wang et al. found that the incidence of PICC-associated VTE in patients with non-O blood types was significantly higher than that in patients with blood group O, which was approximately 2–3.5 times as high as that in patients with blood type O (24). Considering the inconsistency of the results published to date, the current study aimed to conduct a comprehensive meta-analysis of published studies on ABO blood group and PICC-associated VTE susceptibility to provide a more accurate and reliable evidence-based medical basis for the etiology and clinical decision-making regarding PICC-associated VTE susceptibility.

## Materials and Methods

This review was conducted in accordance with the Preferred Reporting Items for Systematic Reviews and Meta-Analyses (PRISMA) guidelines (25).

### Literature Screening and Identification of Relevant Studies

The PubMed, Embase, CNKI, Web of Science, and Wanfang databases were searched for literature on ABO blood groups and the risk of PICC-associated VTE. The language of the included studies was limited to Chinese and English. The databases were searched from inception to December 2021. The search terms included ABO blood group, ABO Blood-Group System, PICC, peripherally inserted central catheters, VTE, and venous thromboembolism.

### Inclusion Criteria

Studies that met the following criteria were included:

- All case–control and cohort studies related to ABO blood type and PICC-associated VTE susceptibility;
- studies that used imaging examination in the identification of PICC-associated VTE;
- sufficient data to calculate the odds ratio (OR) and 95% confidence interval (CI).

### Exclusion criteria

Exclusion criteria were the following:

- Duplicate publications;
- literature with missing or unusable data;
- reviews, meta-analyses, and case series.

### Quality Assessment and Data Extraction

Two authors independently extracted the data. If there was disagreement, consensus was reached through group discussion. The following data were extracted from each study: the first author’s name, year of publication, country of the participants, research type, detection of PICC-associated VTE, total number of participants with PICC-associated VTE per ABO blood type, and Newcastle–Ottawa Scale score.

The Newcastle–Ottawa Scale (NOS) was used to evaluate the quality of observational studies (26). The total scoring system used has ratings ranging from 0 to 9. An accumulated score of ≥7 points indicates high-quality studies.

### Data synthesis and Statistical Analysis

Stata 14.0 (STATA Corp, College Station, TX, USA) was applied for meta-analysis. OR is the statistic of effect size, and each effect size provides its 95% CI. Heterogeneity among the studies was determined using a χ^2^-based Q test, and its degree was measured using *I^2^* statistics. The fixed effects model was used when there was no significant heterogeneity (p>0.1, *I^2^* <50%); otherwise, the random effects model was adopted for meta-analysis. Subgroup analyses were performed based on ethnicity. Sensitivity analysis was conducted by the elimination method one by one to verify the stability of the results of the meta-analysis. Publication bias was assessed by Egger’s regression and Begg’s rank correlation analysis. The significance threshold was set at a two-sided p value of <0.05.

## Results

### Study Identification and Selection

**Figure 1:**
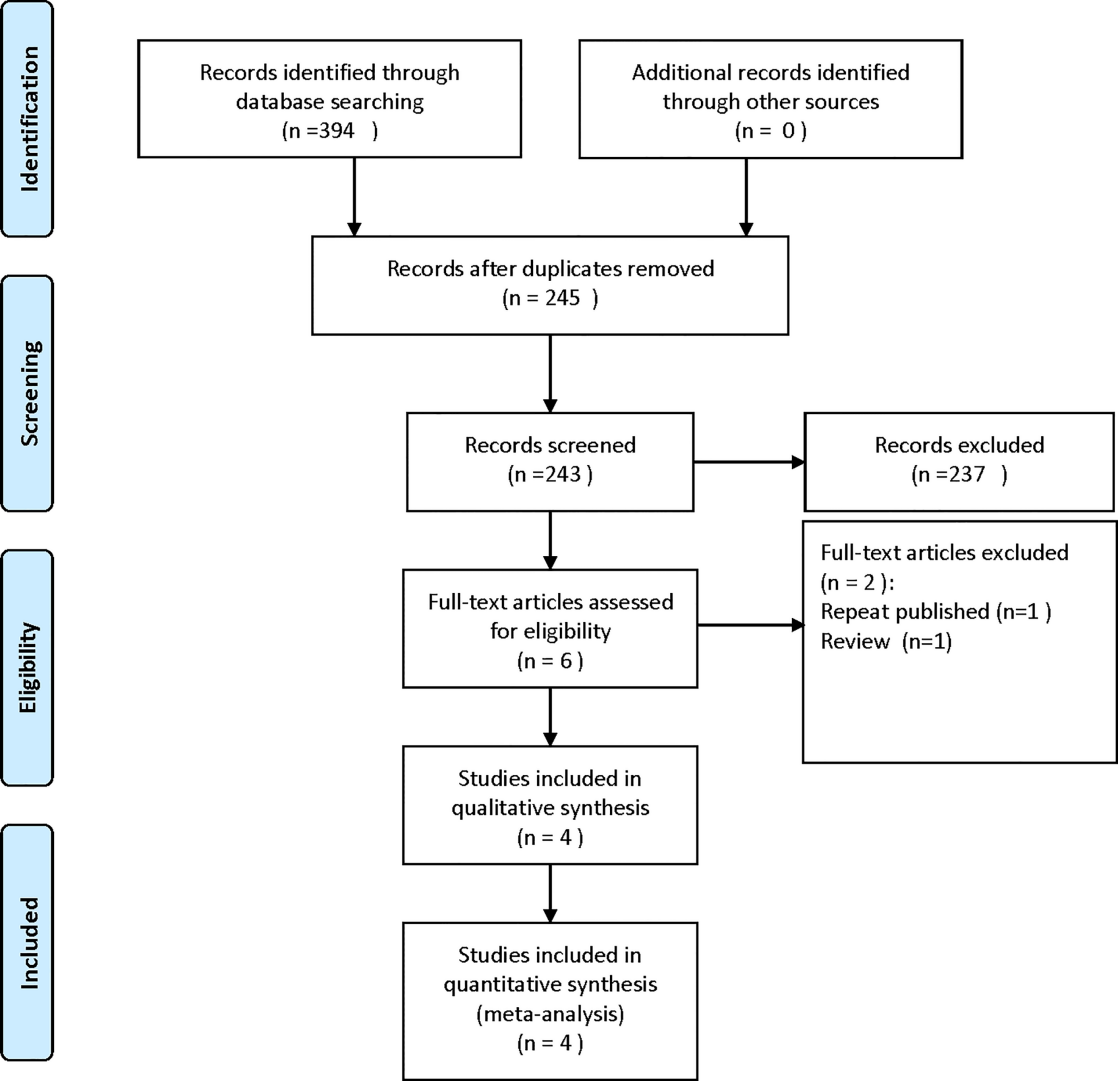
Flow chart of selection process.

A total of 394 articles were obtained by searching relevant electronic databases. A total of 149 duplicates were deleted by Endnote software. After reading the titles and abstracts, 237 references were excluded. Finally, the full text was read, and four studies were included in the meta-analysis. The flow chart of the literature screening process is shown in **Figure 1**.

### Study Characteristics

We included a total of four studies (23, 24, 27, 28) involving 7,804 patients. These studies were conducted between 2010 and 2019. One study (27) was a case–control study. Three of the studies (23, 24, 28) were cohort studies. Two studies (24, 28) were conducted in China, and two others were conducted in the United States (27) and Australia (23). The quality of the included literature was scored 7–8. The general characteristics of the included studies are shown in **Table 1**.

**Table 1:** General characteristics of the included studies.

Author, year	Country	Years	The research type	Testing method	PICC-VTE group				No VTE group				NOS score
					O	A	B	AB	O	A	B	AB	
Haddad et al 2018 (27)	USA	2012-2016	Case control study	Ultrasound	51	44	25	6	35	36	13	3	7
Wang et al 2019 (24)	China	2015-2017	Cohort study	Ultrasound	7	13	25	9	656	577	730	225	8
Koo et al 2017 (23)	Australian	2010-2014	Cohort study	Ultrasound	44	50	22	8	1288	1146	240	122	8
Wang et al 2020 28	China	2018-2019	Cohort study	Ultrasound	21	42	48	20	656	577	726	225	7

NOS, Newcastle-Ottawa Scale; PICC,Peripherally inserted central catheter; VTE, Venous Thromboembolism.

### Association Between O Blood Groups and PICC-Associated VTE

**Figure 2:**
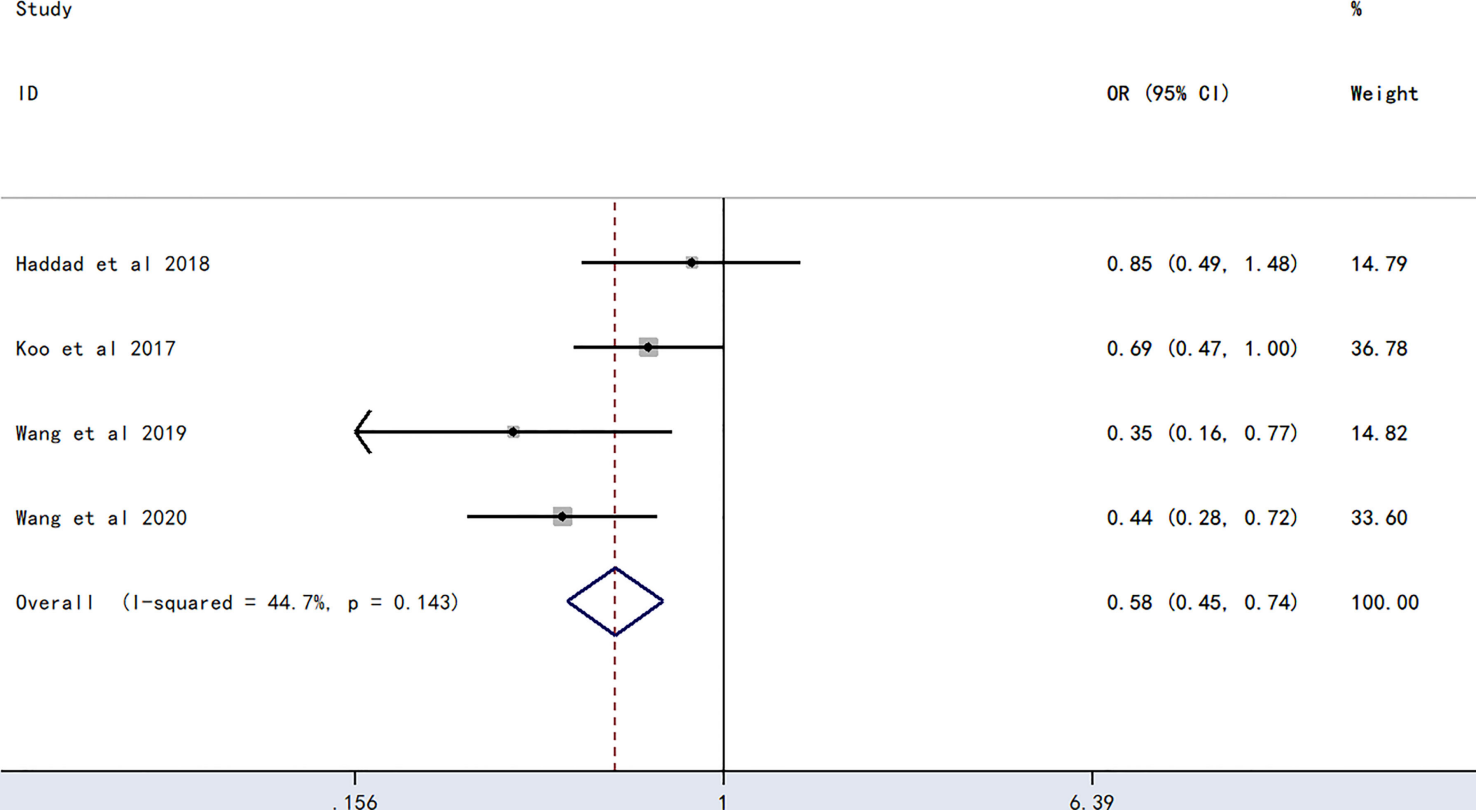
Forest plot evaluating the association between PICC-associated VTE and the prevalence of O blood group.

The meta-analysis of findings from four studies (23, 24, 27, 28) on the association between O blood type and PICC-associated VTE (**Figure 2**) demonstrated lower odds of PICC-associated VTE compared with those of the non-O blood group (OR=0.58, 95% CI=0.45–0.74, p<0.0001).

### Association Between A Blood Groups and PICC-Associated VTE

**Figure 3:**
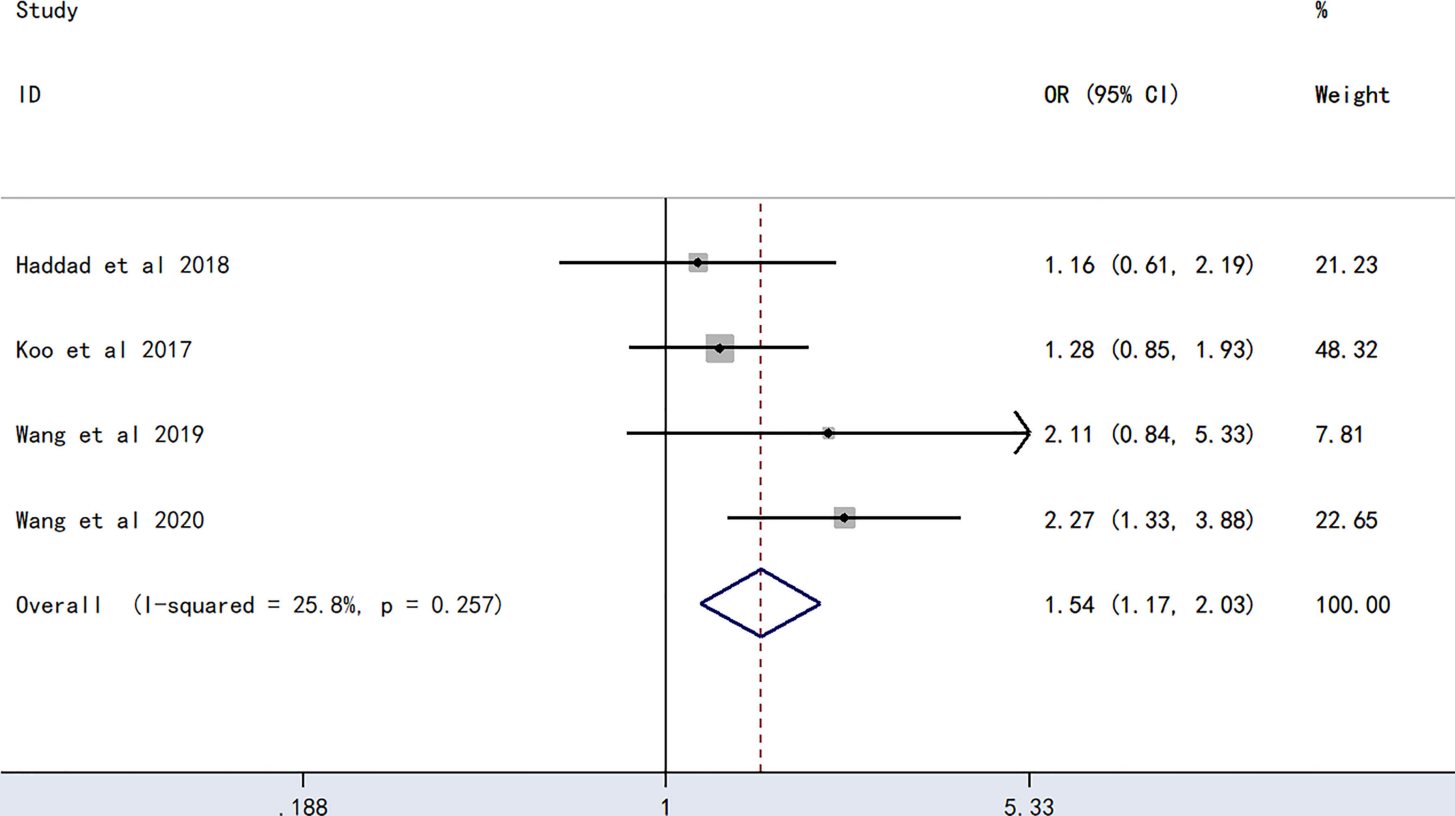
Forest plot evaluating the association between PICC-associated VTE and the prevalence of A blood group.

The meta-analysis of findings from four studies (23, 24, 27, 28) on the association between A blood type and PICC-associated VTE (**Figure 3**) demonstrated higher odds of PICC-associated VTE (OR=1.54, 95% CI=1.17–2.03), p=0.002).

### Association Between B Blood Groups and PICC-Associated VTE

**Figure 4:**
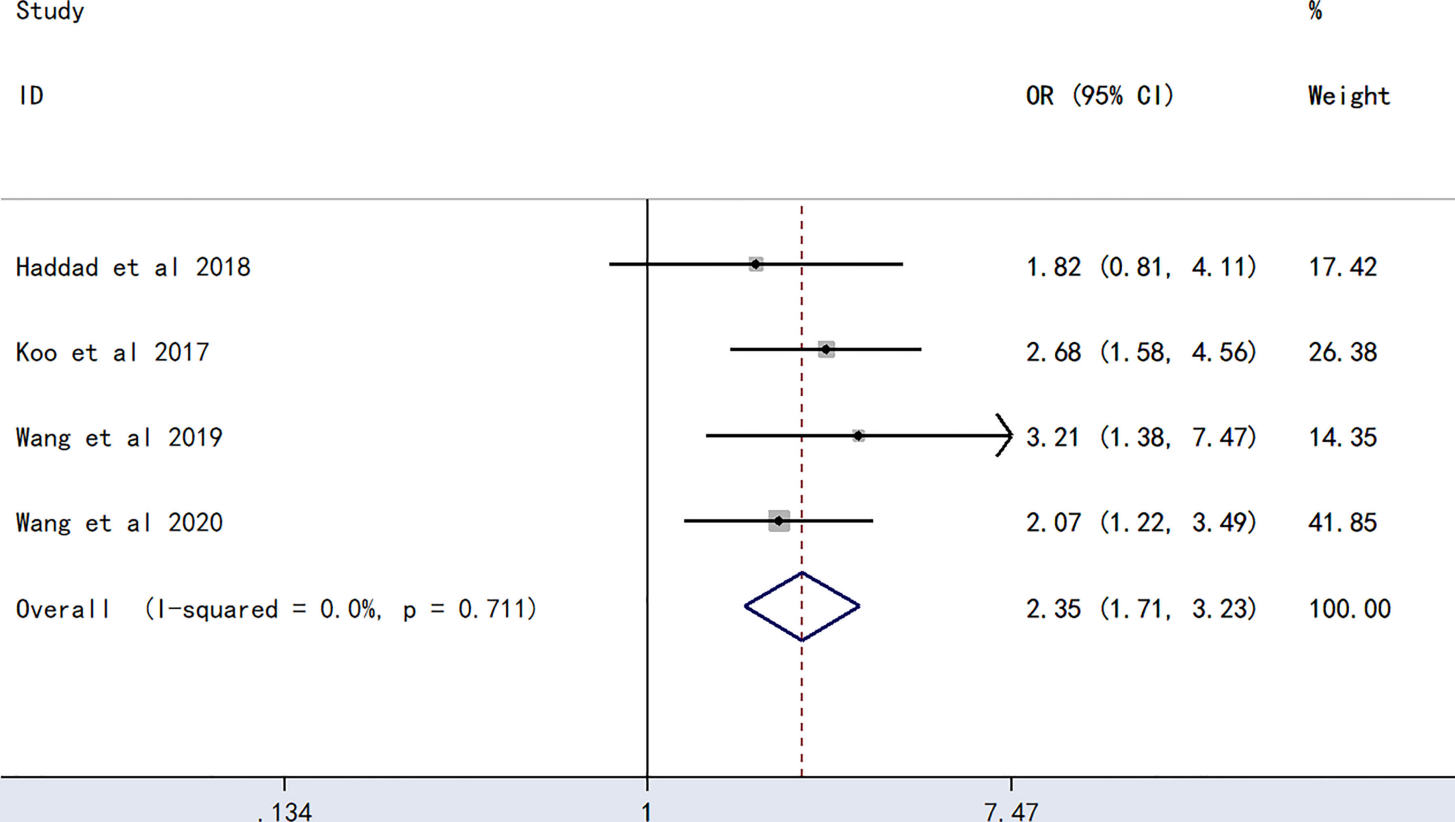
Forest plot evaluating the association between PICC-associated VTE and the prevalence of B blood group.

The meta-analysis of findings from four studies (23, 24, 27, 28) on the association between B blood type and PICC-associated VTE (**Figure 4**) demonstrated higher odds of PICC-associated VTE (OR=2.35, 95% CI=1.71–3.23, p<0.0001).

### Association Between AB Blood Groups and PICC-Associated VTE

**Figure 5:**
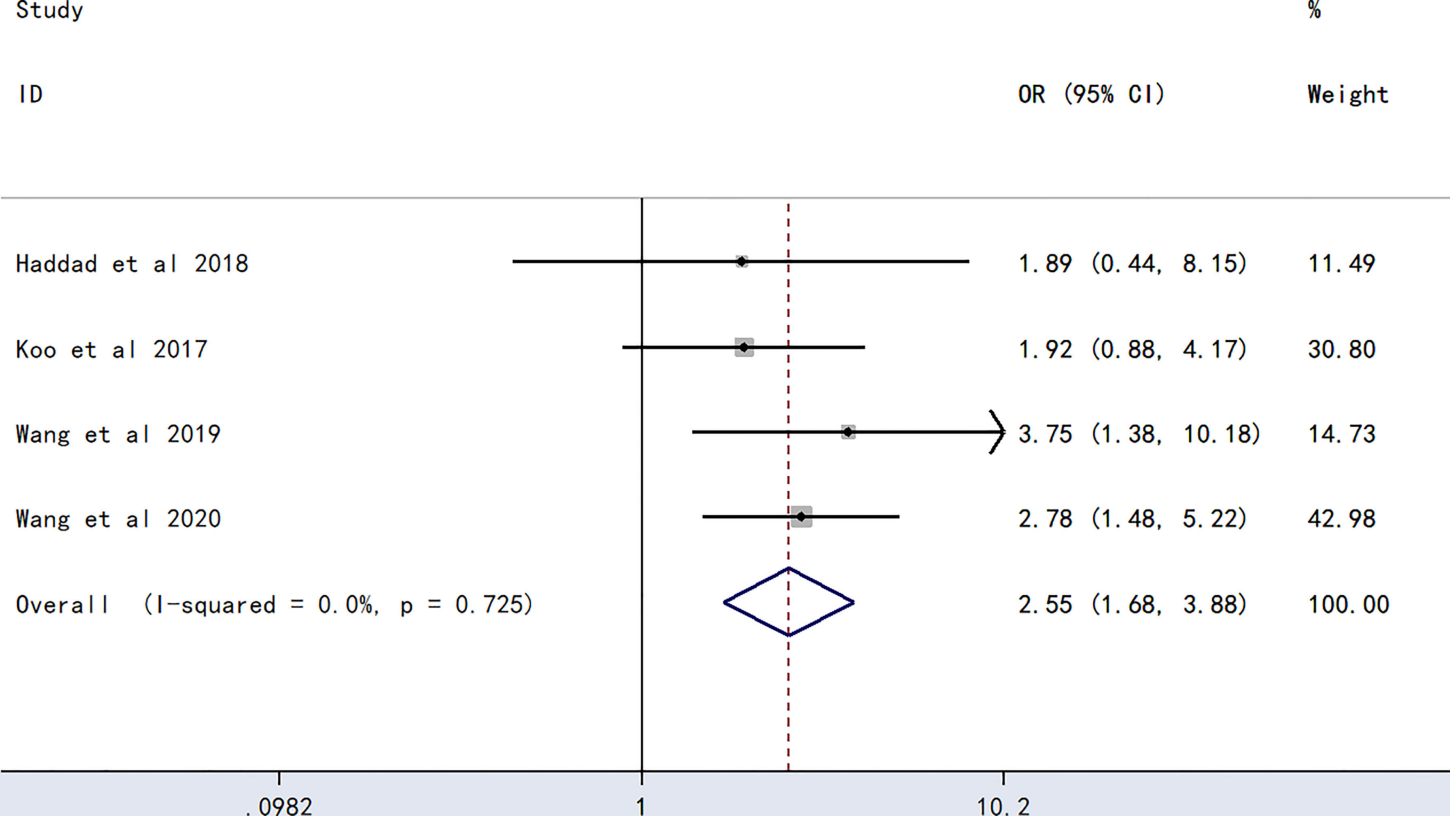
Forest plot evaluating the association between PICC-associated VTE and the prevalence of AB blood group.

The meta-analysis of findings from four studies (23, 24, 27, 28) on the association between AB blood type and PICC-associated VTE (**Figure 5**) demonstrated higher odds of PICC-associated VTE (OR=2.55, 95% CI=1.68–3.88, p<0.0001).

### Subgroup Analysis

We conducted a subgroup analysis based on ethnicity (**Table 2**). Subgroup analysis showed that blood type O was a protective factor against PICC-associated VTE in the Asian population (OR=0.57, 95% CI=0.43–0.76, p<0.0001), while blood types A (OR=1.60, 95% CI=1.15–2.21, p=0.005), B (OR=2.30, 95% CI=1.58–3.37, p<0.0001), and AB (OR=2.42, 95% CI=1.49–3.92, p<0.0001) were risk factors for PICC-associated VTE. Among Caucasians, blood types B (OR=2.45, 95% CI=1.37–4.37, p=0.002) and AB (OR=2.93, 95% CI=1.27–6.76, p=0.011) were risk factors for PICC-associated VTE, while no difference was found in blood types O (OR=0.57, 95% CI=0.24–1.38, p=0.213) and A (OR=1.41, 95% CI=0.84–2.38, p=0.193).

**Table 2:** Results of ethnic subgroup analysis.

Group	Ethnicity	Number of studies	Association test			Heterogeneity		
			OR	95%CI	*p*-val	Model	*p*-val	*I^2^*
O vs Non-O	Overll	4	**0.58**	**(0.45-0.74)**	**<0.0001**	Fixed	0.143	44.70%
	Asian	2	**0.57**	**(0.43-0.76)**	**<0.0001**	Fixed	0.158	49.70%
	Caucasian	2	0.57	(0.24-1.38)	0.213	Random	0.067	70.10%
A vs O	Overll	4	**1.54**	**(1.17-2.03)**	**0.002**	Fixed	0.257	25.80%
	Asian	2	**1.60**	**(1.15-2.21)**	**0.005**	Random	0.094	64.20%
	Caucasian	2	1.41	(0.84-2.38)	0.193	Fixed	0.294	9.30%
B vs O	Overll	4	**2.35**	**(1.71-3.23)**	**<0.0001**	Fixed	0.711	0%
	Asian	2	**2.30**	**(1.58-3.37)**	**<0.0001**	Fixed	0.486	0%
	Caucasian	2	**2.45**	**(1.37-4.37)**	**0.002**	Fixed	0.341	0%
AB vs O	Overll	4	**2.55**	**(1.68-3.88)**	**<0.0001**	Fixed	0.725	0%
	Asian	2	**2.42**	**(1.49-3.92)**	**<0.0001**	Fixed	0.469	0%
	Caucasian	2	**2.93**	**(1.27-6.76)**	**0.011**	Fixed	0.725	0%

Bold indicates that the difference is statistically significant.

### Sensitivity Analysis

**Figure 6:**
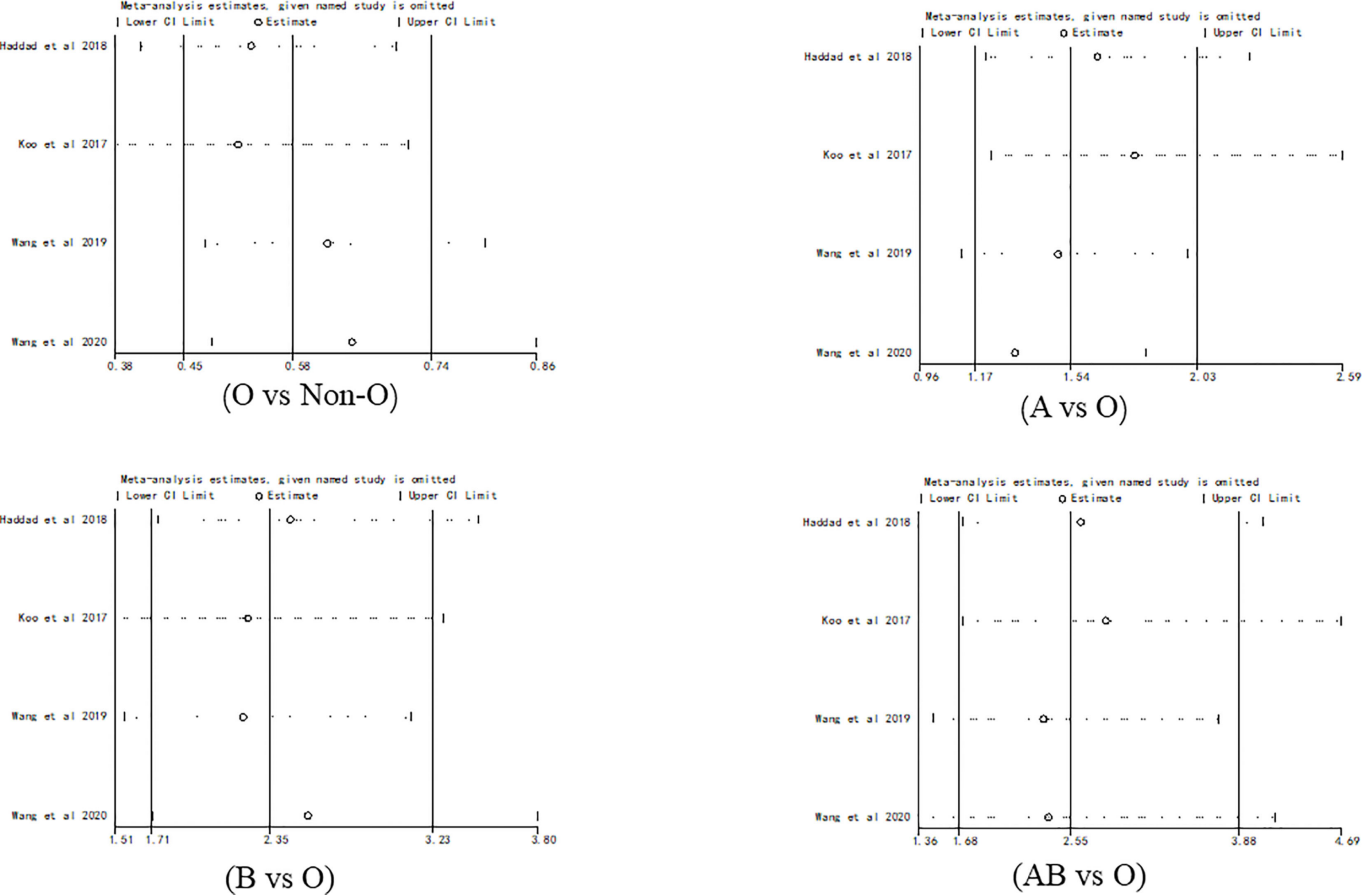
Sensitivity analysis for evaluating the association between PICC-associated VTE and the prevalence of ABO blood group.

Sensitivity analysis showed no outliers (**Figure 6**). Therefore, the results of the meta-analysis were stable.

### Publication Bias

**Figure 7:**
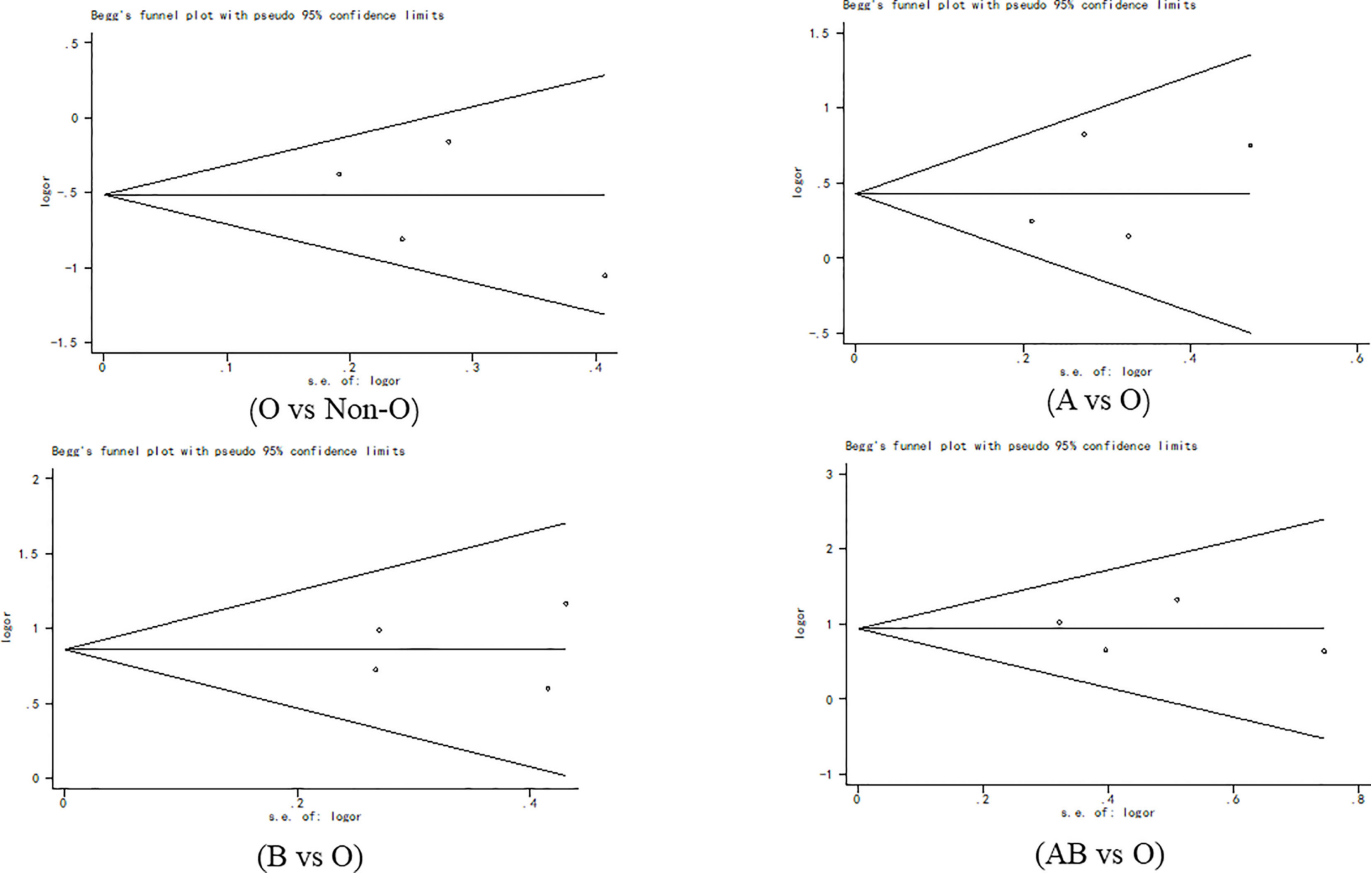
Begg’s funnel plot for publication bias.

Begg’s funnel plot (**Figure 7**) and Egger’s test were used to evaluate publication bias. The results showed no statistically significant difference (O vs. Non-O: Begg’s test p = 1.000, Egger’s test p = 0.518; A vs. O: Begg’s test p = 0.497, Egger’s test p =0.628; B vs. O: Begg’s test p = 0.497, Egger’s test p =0.899; AB vs. O: Begg’s test p = 1.000, Egger’s test p =0.878).

## Discussion

The results of the pooled data analysis indicated that O blood type was a protective factor against PICC-associated VTE compared with non-O blood type, especially in Asian populations. Additionally, blood types A, B, and AB were risk factors for PICC-associated VTE. To our knowledge, this is the largest and possibly the only study reporting an association between ABO blood group and the risk of PICC-associated VTE.

Various factors, including genetic and environmental factors, contribute to the development of VTE. Although the exact causal mechanism is not known, the presence of a prothrombotic state may be considered the most likely factor linking VTE to ABO blood types (29). In fact, non-O blood type subjects have higher levels of vWF and circulating factor VIII than O blood type subjects (30). Therefore, this is recognized as a risk factor for thrombosis. Many studies have determined the causal role of hypercoagulable states on the risk and severity of thrombosis (31). The meta-analysis of Dentali et al. (22) found that the risk of thrombosis was significantly higher among individuals with non-O blood types than those with O blood type. The findings confirm the higher risk of pulmonary embolism and deep vein thrombosis in non-O blood type (32) and that individuals with non-O blood types are at higher risk for PE and DVT (33).

Vasan et al. (32) conducted a study of 1.5 million blood donors and confirmed a doubling of the risk of different thromboembolisms in patients with non-O blood types. However, fewer studies have been conducted on the relationship between ABO blood group and PICC-associated VTE, and the results have been inconsistent. Whether ABO blood group can be considered a risk factor for PICC-associated VTE lacks confirmation from large sample studies. This meta-analysis found for the first time a significantly higher incidence of PICC-associated VTE in patients with non-O blood groups than in patients with O blood type. When subgroup analyses of ethnicity were performed, the most impressive results were that in Asian populations, blood type O may be a genetic protective factor for PICC-associated VTE, and blood types A, B, and AB are risk factors for PICC-associated VTE. Among Caucasians, blood types B and AB had a higher risk of PICC-associated VTE. This may be due to the higher percentage of non-O blood types in Asian populations compared to Caucasians. A hospital-based study of 200,000 patients showed that the proportion of non-O blood types in the Chinese Han race was approximately 70% (34). Several other studies have reported approximately 50%–60% non-O blood in Caucasians (21, 32, 35, 36). The Chinese population may have a higher rate of VTE because non-O blood type is a risk factor. Therefore, the results of this meta-analysis may be more appropriate for Asian populations, especially Chinese populations.

Both genetic and environmental factors contribute to the development of VTE. Among the genetic factors, ABO blood type has a profound effect on hemostasis and is presumed to be associated with VTE. People with O blood type have lower plasma levels of circulating factor VIII and vWF, which may protect them from VTE. Schleef et al. (37) showed that vWF levels in the O-group population ranged from 654 to 1,028 U/L, whereas in the non-O-group population, their vWF levels ranged from 900 to 1,390 U/L, with a mean level of 1,339 U/L. Plasma vWF and circulating factor VIII concentrations are approximately 25% higher in non-O patients than in O patients (38). vWF is a multimer of variable molecular weight secreted by endothelial cells and megakaryocytes (39). It can promote thrombosis by mediating the adhesion reaction of platelets to subendothelial collagen, mediating platelet-to-platelet aggregation, and acting as a protective carrier of coagulation factor VIII (40, 41). PICC-related VTE is caused by multiple factors and pathways, including exogenous and endogenous pathways of thrombosis. Catheters act as intravascular foreign bodies, and when endothelial cells are injured, collagen under the exposed endothelium activates platelets and coagulation factor XII, initiating the endogenous coagulation process (42). Additionally, the damaged endothelial cells release tissue factor, which activates coagulation factor VII, thus initiating the exogenous coagulation process, activating the coagulation system, and eventually leading to thrombosis (43). Plasma vWF levels are elevated during vascular endothelial injury and participate in thrombus formation by mediating platelet adhesion (44). Higher plasma vWF and circulating factor VIII concentrations in the non-O-group population promote PICC-related VTE formation. Therefore, in clinical practice, physicians should be aware of non-O blood types as risk factors for PICC-related VTE.

## Limitations

The results of this study suggested that ABO blood groups may be associated with susceptibility to PICC-associated VTE. However, attention should be given to the limitations of this study when interpreting these results. First, due to the limitation of the number of studies, more ethnic groups were not included for subgroup analysis. Therefore, there is little evidence suggesting that the results of this study are applicable to other ethnic groups. Second, the included studies only included literature published in Chinese and English. Additionally, the objective effect of ABO blood group on PICC-associated VTE susceptibility may be affected by correcting for gender, age, smoking, and other patient characteristics. Considering these limitations, the results of this meta-analysis should be treated with caution for clinical application.

## Conclusions

In conclusion, our meta-analysis found that O blood type is a protective factor against PICC-associated VTE, especially in the Asian population. Additionally, blood types A, B, and AB are risk factors for PICC-associated VTE. Further functional studies are recommended to determine the exact role of ABO blood groups in the development of PICC-associated VTE.

## Data Availability Statement

The datasets presented in this study can be found in online repositories. The names of the repository/repositories and accession number(s) can be found in the article/supplementary material.

## Author Contributions

Conceptualization: QZ and JS. Data collection: HP, LH, and RR. Funding acquisition: JS. Resources: JS. Software: HP and LH. Supervision: JS. Writing–original draft: QZ, XP, and JS. Writing—review and editing: QZ, XP, and JS.

## Conflict of Interest

The authors declare that the research was conducted in the absence of any commercial or financial relationships that could be construed as a potential conflict of interest.

## Publisher’s Note

All claims expressed in this article are solely those of the authors and do not necessarily represent those of their affiliated organizations, or those of the publisher, the editors and the reviewers. Any product that may be evaluated in this article, or claim that may be made by its manufacturer, is not guaranteed or endorsed by the publisher.
